# When and how do patients with cardiac amyloidosis die?

**DOI:** 10.1007/s00392-019-01490-2

**Published:** 2019-05-27

**Authors:** F. Escher, M. Senoner, J. Doerler, M. M. Zaruba, M. Messner, C. Mussner-Seeber, M. Ebert, C. Ensinger, A. Mair, A. Kroiss, H. Ulmer, S. Schneiderbauer-Porod, C. Ebner, G. Poelzl

**Affiliations:** 1 Clinical Division of Cardiology and Angiology, Medical University of Innsbruck, Anichstrasse 35, 6020 Innsbruck, Austria; 2 Department of Cardiology, LMU Munich, Munich, Germany; 3 Department of Pathology, Medical University of Innsbruck, Innsbruck, Austria; 4 Department of Radiology, Medical University of Innsbruck, Innsbruck, Austria; 5 Department of Nuclear Medicine, Medical University of Innsbruck, Innsbruck, Austria; 6 Department of Medical Statistics, Informatics and Health Economics, Medical University of Innsbruck, Innsbruck, Austria; 7 Department of Cardiology, Ordensklinikum Elisabethinen Linz, Linz, Austria

**Keywords:** Cardiac amyloidosis, Light chain (AL) amyloidosis, Transthyretin (ATTR) amyloidosis, Prognosis, Mode of death

## Abstract

**Background:**

Cardiac amyloidosis (CA) is an underappreciated cause of morbidity and mortality. Light-chain (AL) and transthyretin (ATTR) amyloidosis have different disease trajectories. No data are available on subtype-specific modes of death (MOD) in patients with CA.

**Methods and results:**

We retrospectively investigated 66 with AL and 48 with wild-type ATTR amyloidosis (ATTRwt) from 2000 to 2018. ATTRwt differed from AL by age (74.6 ± 5.4 years vs. 63 ± 10.8 years), posterior wall thickness (16.8 ± 3.3 mm vs. 14.3 ± 2.2 mm), left ventricular mass index (180.7 ± 63.2 g/m^2^ vs. 133.5 ± 42.2 g/m^2^), and the proportions of male gender (91.7% vs. 59.1%), atrial enlargement (92% vs. 68.2%) and atrial fibrillation (50% vs. 12.1%). In AL NYHA Functional Class and proteinuria (72.7% vs. 39.6%) were greater; mean arterial pressure (84.4 ± 13.5 mmHg vs. 90.0 ± 11.3 mmHg) was lower. Unadjusted 5-year mortality rate was 65% in AL-CA vs. 44% in the ATTRwt group. Individuals with AL-CA were 2.28 times ([95%CI 1.27–4.10]; *p* = 0.006) more likely to die than were individuals with ATTRwt-CA. Information on MOD was available in 56 (94.9%) of 59 deceased patients. MOD was cardiovascular in 40 (66.8%) and non-cardiovascular in 16 (27.1%) patients. Cardiovascular [28 (68.3%) vs. 13 (80%)] death events were distributed equally between AL and ATTRwt (*p* = 0.51).

**Conclusion:**

Our data indicate no differences in MOD between patients with AL and ATTRwt cardiac amyloidosis despite significant differences in clinical presentation and disease progression. Cardiovascular events account for more than two-thirds of fatal casualties in both groups.

**Graphic abstract:**

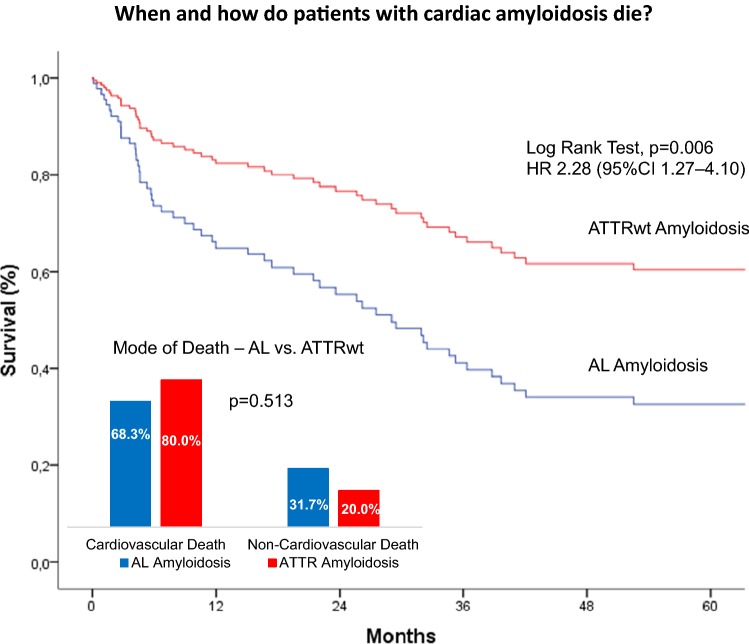

## Introduction

Cardiac amyloidosis is an infiltrative process of the extracellular matrix that increases myocardial wall thickness in the absence of actual cardiomyocyte hypertrophy [1]. Acquired monoclonal immunoglobulin light-chain amyloidosis (AL), the hereditary, transthyretin (TTR)-related form (ATTRm), and wild-type (non-mutant) TTR-related amyloidosis (ATTRwt) systemic “senile” amyloidosis account for more than 90% of all cardiac amyloidosis (CA) [2].

While AL is considered a rare disease [3] and ATTRm is mostly seen in endemic regions [4] and in elderly patients of African descent [5], recent evidence suggests that ATTRwt is probably much more common than widely appreciated. ATTR was evident in patients with heart failure with preserved ejection fraction (HFpEF) [6] and in elderly patients with aortic stenosis [10]. In Finland, ATTR was found in 25% of autopsies in very old persons [11], giving rise to the suspicion that ATTRwt could be the most frequent form of CA [9].

AL typically affects multiple organ systems. Cardiac involvement is found in up to 70% of cases. While frequency of cardiac amyloidosis (CA) in ATTRm is variable and depends on the specific mutation [12], ATTRwt almost exclusively affects the heart. CA is typically associated with heart failure and dictates the clinical course of the disease. Most importantly, disease profiles and clinical courses differ between AL and ATTR [2, 13]. Prognosis is poor in patients with CA with better survival in ATTR than in AL [2, 8, 13, 14]. Also, the two subtypes of CA differ significantly with respect to treatment options [8, 15].

A major limitation observed in the previously published literature is the fact that despite the commonly held notion that death in CA occurs either as a result of progressive heart failure or sudden cardiac death [16], mode of death in patients with cardiac AL and TTR amyloidosis has hardly been studied so far [17].

It was the aim of this comprehensive retrospective study to provide data on disease progression and mode of death in patients with cardiac AL and TTR amyloidosis to improve clinical management and service provision in these patients.

## Methods

The study cohort consisted of consecutive patients with confirmed AL or ATTRwt amyloidosis seen between May 2000 and June 2018 at a tertiary (Cardiology Department, Medical University of Innsbruck) and a secondary (Cardiology Department, Ordensklinikum Elisabethinen Linz) centre. A comprehensive baseline assessment was performed in all patients including initial clinical evaluation and follow-up as well as laboratory, electrocardiographic, and echocardiographic parameters. Informed consent was waived due to the retrospective nature of the trial. The study was approved by the ethics committee of the Medical University of Innsbruck.

### Diagnostic definition

Diagnosis of systemic amyloidosis was defined by histological documentation of Congo Red staining and apple-green birefringence under cross-polarized light in at least one involved organ. Cardiac amyloidosis was diagnosed either by means of endomyocardial biopsy (EMB), cardiac imaging [echocardiography, cardiac magnetic resonance (CMR) or 99mTC-3,3-diphosphono-1,2-propanodicarboxyl acid (99mTC-DPD) scintigraphy] and/or elevation of biomarkers (N-terminal pro brain natriuretic peptide, troponin T) in patients with a positive result of non-cardiac biopsy [2, 18–20].

Diagnosis of ATTRm was defined by a documented TTR mutation at DNA analysis, ATTRwt by positive immunohistochemistry for TTR in the absence of any TTR mutation and AL by the presence of monoclonal plasma cells in the bone marrow. The Mayo staging system was used for risk stratification in AL and TTR amyloidosis using different cut-off values for troponin T and NT-proBNP for each subtype [21, 22].

### Data collection and definitions

Follow-up was closed in June 2018. For patients who had not attended a follow-up in the last 3 months, vital status was ascertained by telephone contact and/or by contacting referring physicians. Information on death was retrieved from patients’ charts, family doctors and relatives, and official documents of death. All deaths were adjudicated by two senior cardiologists (C. E. and G. P.). We employed a classification system derived from the “ACME system” for death in heart failure [23]. Mode of death (MOD) was first categorized as cardiovascular (CV) or non-cardiovascular (non-CV). CV deaths were subsequently classified as cardiac (sudden cardiac death [SCD] or circulatory failure) or vascular (stroke and “other” CV deaths including peripheral vascular disease, pulmonary embolism, mesenteric infarction, and procedural complications). SCD and circulatory failure comprising cardiogenic shock, pulmonary oedema and acute heart failure were defined according to established criteria [24].

### Statistical analysis

Continuous data were tested for normal distribution using the Kolmogorov–Smirnov test. Categorical variables are presented as percentage (%), continuous variables as mean [standard deviation (SD)] or median (25th, 75th percentile). Between-group comparisons were performed with the T test, Mann–Whitney *U* test or Pearson’s Chi-squared test, as appropriate.

Survival analysis was performed for the AL and the ATTRwt amyloid subtypes with all-cause mortality as the endpoint. Patients were censored on June 30, 2018. The unadjusted association between amyloid subtypes and mortality was assessed using a univariable Cox proportional hazards model. Hazard ratios, 95% CIs and *p* values were calculated. A multivariable Cox model adjusted for age and sex was developed by selecting variables that were clinically relevant. Cardiovascular and non-cardiovascular modes of death were compared between groups with Fisher’s exact test.

A two-sided *p* value of 0.05 was considered to be statistically significant. All calculations were performed using the SPSS statistical package, version 23.0 (SPSS Inc., Chicago, IL, USA).

## Results

**Fig. 1:**
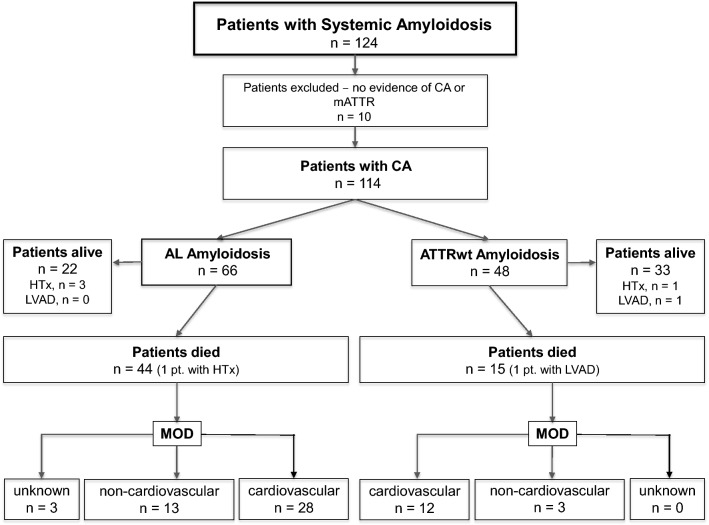
Assembly of the cohort, participant flow, and mode of death in deceased patients. *CA* cardiac amyloidosis, *HTx* heart transplantation, *LVAD* left ventricular assist device, *MOD* mode of death

A total of 124 patients with amyloidosis were screened for inclusion (Fig. 1). Three patients were excluded because of a transthyretin mutation; in seven patients, cardiac involvement could not be demonstrated. The final study cohort consisted of 114 patients: 66 with AL and 48 with ATTRwt amyloidosis. Diagnosis in ATTRwt was based on EMB in 94% and 99mTC-DPD scintigraphy in 6% of patients, and in AL patients on EMB in 62.1% and on non-cardiac biopsy in 37.9%. In those in whom the diagnosis was reached by non-cardiac biopsy, definition of cardiac involvement was based on echocardiography and/or CMR plus elevations of biomarkers.

AL amyloidosis was associated with multiple myeloma in 41 (62%) and Waldenström’s macroglobulinemia in 25 (38%) patients.

Baseline characteristics of AL vs. ATTRwt for the total population are shown in Table 1.

**Table 1:** Patient baseline and treatment characteristics

	All Patients (*n* = 114)	AL (*n* = 66)	ATTRwt (*n* = 48)	*p* value
Demographics				
Sex (male)	83 (72.8)	39 (59.1)	44 (91.7)	< 0.001
Age (years)	67.8 ± 10.6	63.0 ± 10.8	74.6 ± 5.4	< 0.001
BMI (kg/m^2^)	25.1 ± 3.9	24.5 ± 4.1	25.9 ± 3.5	0.054
Diagnosis of CA				
Echocardiography	114 (100)	66 (100)	48 (100)	1.000
Cardiac MRI	81 (71.1)	44 (66.7)	39 (76.4)	0.509
DPD-Tc scintigraphy	14 (12.3)	5 (7.6)	9 (18.8)	0.088
Endomyocardial biopsy	86 (75.4)	41 (62.1)	45 (93.75)	< 0.001
Extracardiac biopsies	25 (21.9)	25 (37.9)	0 (0)	< 0.001
Cardiac characteristics				
NYHA functional class				0.047
I	11 (9.7)	6 (9.1)	5 (10.4)	
II	51 (44.7)	24 (36.4)	27 (56.3)	
III/IV	52 (45.6)	36 (54.5)	16 (33.3)	
Mayo staging score^a^				0.547
I	3 (3.3)	2 (3.5)	1 (2.9)	
II	23 (25.0)	12 (21.1)	11 (31.4)	
III	66 (71.7)	43 (75.4)	23 (65.7)	
MAP (mmHg)	86.7 ± 12.9	84.4 ± 13.5	90.0 ± 11.3	0.018
CAD*	20 (17.7)	10 (15.2)	10 (20.8)	0.458
Valvular heart disease^b^	11 (9.6)	8 (12.1)	3 (6.3)	0.352
NTpro-BNP (ng/L)	4578 ± 4378	5141 ± 5300	3873 ± 2730	0.543
Cardiac troponin T (ng/L)^c^	77.6 ± 70.4	86.8 ± 84.4	62.3 ± 32.3	0.673
Echocardiography				
Atrial enlargement	88 (77.2)	45 (68.2)	43 (89.6)	0.005
LV-EF (%)	51.5 ± 11.4	53.6 ± 11.4	48.7 ± 11.1	0.020
PWD, mm	15.4 ± 3.0	14.3 ± 2.2	16.8 ± 3.3	< 0.001
LV mass (g)	281.3 ± 108.9	247.0 ± 90.6	331.0 ± 114.8	< 0.001
LV mass index (g/m^2^)	152.4 ± 56.3	133.5 ± 42.2	180.7 ± 63.2	< 0.001
E/A ratio	1.81 ± 1.22	1.68 ± 1.04	2.15 ± 1.64	0.457
Pericardial effusion	38 (33.3)	24 (36.4)	14 (29.2)	0.220
ECG				
Atrial fibrillation	32 (28.1)	8 (12.1)	24 (50.0)	< 0.001
First-degree AV-block	24 (21.1)	7 (10.6)	17 (35.4)	0.002
Third-degree AV-block	3 (2.6)	2 (3.0)	1 (2.1)	1.0
Low voltage signs	23 (20.2)	18 (27.3)	5 (10.4)	0.032
Pseudoinfarct pattern	29 (25.4)	15 (22.7)	14 (29.2)	0.520
Renal characteristics				
Creatinine (mg/dl)	1.26 ± 0.71	1.24 ± 0.90	1.28 ± 0.29	0.008
eGFR (ml/min)	62.4 ± 22.4	66.8 ± 25.6	56.3 ± 15.4	0.007
Proteinuria	67 (58.8)	48 (72.7)	19 (39.6)	0.001
Specific therapy				
Immunotherapy		53 (80.3)		n/a
Chemotherapy		56 (84.0)		n/a
ASCT		14 (21.2)		n/a
Green tea capsules/EGCG	23 (20.2)	6 (9.1)	17 (35.4)	< 0.001
Tafamidis			3 (5.9)	n/a
Anticoagulant therapy	75 (65.8)	40 (60.6)	35 (72.9)	0.158
ICD	11 (9.6)	4 (6.1)	7 (14.6)	0.198
Pacemaker	18 (15.8)	8 (12.1)	10 (20.8)	0.298
HTx/LVAD	5 (4.4)	3 (4.5)	2 (4.2)	0.637

Data from 114 patients are reported as mean (± standard deviation) or number (percentage)

*BMI* body mass index, *NYHA* New York heart association, *MAP* mean atrial pressure pressure, *CAD* coronary artery disease, *NT-proBNP* N-terminal pro-B-type natriuretic peptide, *LV-EF* left ventricular ejection fraction, *Syst*. *PWD* posterior wall thickness, *eGFR* estimated glomerular filtration rate, *ASCT* autologous stem cell transplantation, *EGCG* epigallocatechin gallate, *ICD* implantable cardioverter/defibrillator, *HTx* heart transplantation, *LVAD* left ventricular assist device

^a^≥ 70% stenosis a/o need for coronary intervention

^b^Including severe aortic stenosis, mitral regurgitation/stenosis, and tricuspid regurgitation

^c^Troponin T and Mayo staging score were available in 92 patients-different Mayo staging scores were applied for each subtype according to Dispenzieri et al. [21] and Grogan et al. [22]

### Mortality and mode of death

Overall, 325.7 patient years of follow-up were registered. Median follow-up for the entire cohort was 21.1 months (5.6–54.4). The group of patients with an AL subtype had a median follow-up of 16 months (4.6–60.9), while the ATTRwt cohort had a median follow-up of 24.6 months (6.8–50.6) (*p* = 0.81).

After censoring follow-up, 59 (51.8%) of all patients met the endpoint of all-cause mortality. Of the patients with AL amyloidosis 44 died, corresponding to an unadjusted 5-year mortality rate of 65%, while in the ATTRwt group there were 15 deaths, corresponding to a 5-year mortality rate (25.0% 30-month mortality rate). Four patients received a heart transplant, three in the AL and one in the ATTRwt group, and one patient with TTR amyloidosis a LV assist device over the course of the study period.

Baseline characteristics of non-survivors vs. survivors are shown in Table 2. Deceased patients presented with higher NT-proBNP levels (*p* = 0.003) and higher NYHA functional class (*p* = 0.039). Interestingly, the proportion of patients in atrial fibrillation was significantly larger in survivors (*p* = 0.023). The Mayo score was not different between groups (*p* = 0.287). This was also true when AL and ATTRwt patients were analysed separately (*p* = 0.154 and *p* = 0.764, respectively).

**Table 2:** Baseline characteristics in survivors vs. non-survivors

	Survivors (*n* = 55)	Non-survivors (*n* = 59)	*p* value
Demographic characteristics			
Sex (male)	42 (76.4)	41 (69.5)	0.528
Age (years)	68.2 ± 11.3	67.5 ± 10.0	0.378
BMI (kg/m^2^)	25.3 ± 3.9	24.9 ± 3.8	0.605
Cardiac characteristics			
NYHA functional class			0.039
I	8 (14.6)	3 (5.1)	
II	28 (50.9)	23 (39.0)	
III/IV	19 (34.5)	33 (55.9)	
Mayo staging score^a^			0.287
I	3 (6.4)	0 (0.0)	
II	12 (25.5)	11 (24.4)	
III	32 (68.1)	34 (75.6)	
MAP (mmHg)	88.4 ± 10.5	85.2 ± 14.6	0.192
CAD*	12 (21.8)	7 (11.9)	0.324
Valvular heart disease^b^	4 (7.3)	8 (13.6)	0.364
NTpro-BNP (ng/L)	3569 ± 3232	5623 ± 5137	0.003
Cardiac troponin T (ng/L)^c^	72.5 ± 61.5	82.6 ± 78.8	0.665
Echocardiography			
Atrial enlargement	39 (70.1)	49 (83.1)	0.181
LV-EF	52.9 ± 11.1	50.2 ± 11.6	0.235
PWD (mm)	15.7 ± 3.5	15.1 ± 2.5	0.307
LV Mass (g)	301.0 ± 127.0	266.0 ± 90.0	0.121
LV mass Index (g/m^2^)	163.5 ± 67.5	143.9 ± 44.7	0.179
E/A ratio	1.40 ± 0.89	2.05 ± 1.34	0.093
Pericardial effusion	18 (32.7)	20 (33.9)	0.686
ECG			
Atrial fibrillation	21 (38.2)	11 (18.6)	0.023
AV-block	15 (27.3)	12 (20.3)	0.508
First-degree AV-block	14 (25.4)	10 (16.9)	0.358
Third-degree AV-block	1 (1.8)	2 (3.4)	1.0
Pseudoinfarct patterns	15 (27.3)	14 (23.7)	0.673
Renal characteristics			
Creatinine (mg/dl)	1.27 ± 0.90	1.25 ± 0.48	0.725
eGFR (ml/min)	63.8 ± 23.7	61.1 ± 21.3	0.531
Proteinuria	28 (50.1%)	39 (66.1%)	0.241
Specific therapy			
Green tea capsules/EGCG	10 (18.2)	13 (22.0)	0.645
Anticoagulant therapy	35 (63.6)	40 (67.8)	0.842
ICD	6 (10.9)	5 (8.5)	0.756
Pacemaker	5 (9.1)	13 (22.0)	0.074
HTx/LVAD	3 (5.5)	2 (3.4)	0.673

Data from 114 patients are reported as mean (± standard deviation) or number (percentage)

*BMI* body mass index, *NYHA* New York heart association, *MAP* mean atrial pressure pressure, *CAD* coronary artery disease, *NT-proBNP* N-terminal pro-B-type natriuretic peptide, *LV-EF* left ventricular ejection fraction, *Syst.; PWD* posterior wall thickness, *eGFR* estimated glomerular filtration rate, *EGCG* epigallocatechin gallate, *ICD* implantable cardioverter/defibrillator, *HTx* heart transplantation, *LVAD* left ventricular assist device

^a^≥ 70% stenosis a/o need for coronary intervention

^b^Including severe aortic stenosis, mitral regurgitation/stenosis, and tricuspid regurgitation

^c^Troponin T and Mayo staging score were available in 92 patients

**Fig. 2:**
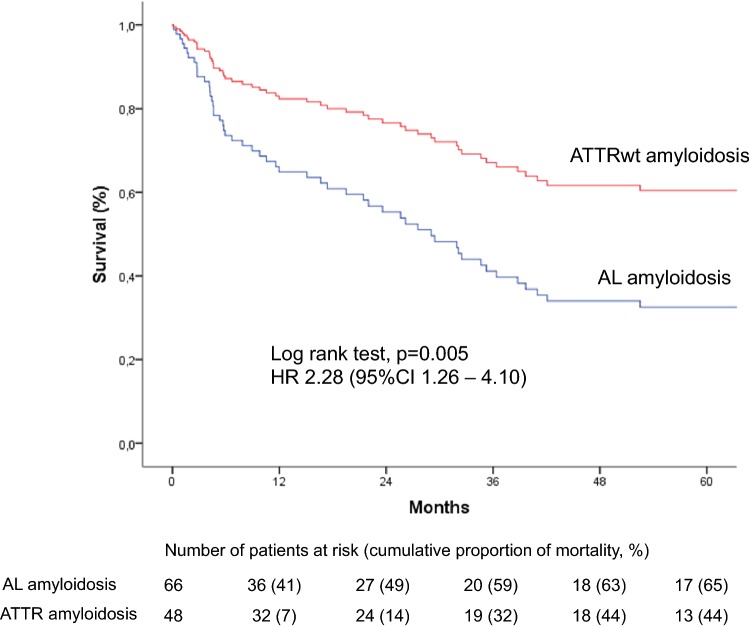
Correlation between cardiac AL and TTR amyloidosis and mortality Cumulative 5-year event rates estimated by univariate Cox proportional hazard regression analysis in 117 patients with cardiac amyloidosis according to subtype are presented. Numbers of patients at risk and event rates are shown below the graphs

Cox regression analysis showed individuals with AL amyloidosis to be 2.28 times ([95%CI 1.27–4.10]; *p* = 0.006) more likely to die than were individuals with ATTRwt amyloidosis (Fig. 2). Likewise, the combined endpoint of death, heart transplantation or LV assist device implantation was significantly higher in AL patients (HR 2.01 [95%CI 1.22–3.83]; *p* = 0.008). Multivariate Cox regression analysis adjusted for sex and age demonstrated that AL amyloid-specific subtype and lnNT-proBNP were associated with mortality independent of LV-EF, MAP, NYHA functional class, atrial fibrillation, and eGFR (Table 3). Results remained robust when in a subgroup of patients (*n* = 92) the Mayo staging score was added to the model.

**Table 3:** Association between amyloidosis subtypes and survival during the observation period using multivariate, sex- and age-adjusted Cox proportional hazards regression analyses

	Multivariate model adjusted for age and sex			
	Wald	HR	95% CI	*p* value
AL vs. ATTRwt	7.02	3.03	1.33–6.87	0.008
LV-EF, per %	1.47	0.98	0.96–1.01	0.225
MAP, per mmHg	0.75	0.99	0.97–1.01	0.387
NYHA class, overall	0.64			0.728
A-Fib, y/n	0.59	0.71	0.30–1.67	0.441
lnNT-proBNP, per ln ng/L	11.95	2.16	1.39–3.33	0.001
eGFR, per ml/min/1.73 m^2^	2.467	1.02	0.99–1.02	0.116

*LV-EF* left ventricular ejection fraction, *NYHA* New York Heart Association, *MAP* mean atrial pressure, *A-Fib* atrial fibrillation, *lnNT-proBNP* logarithmically transformed N-terminal pro-B-type natriuretic peptide, *eGFR* estimated glomerular filtration rate

**Fig. 3:**
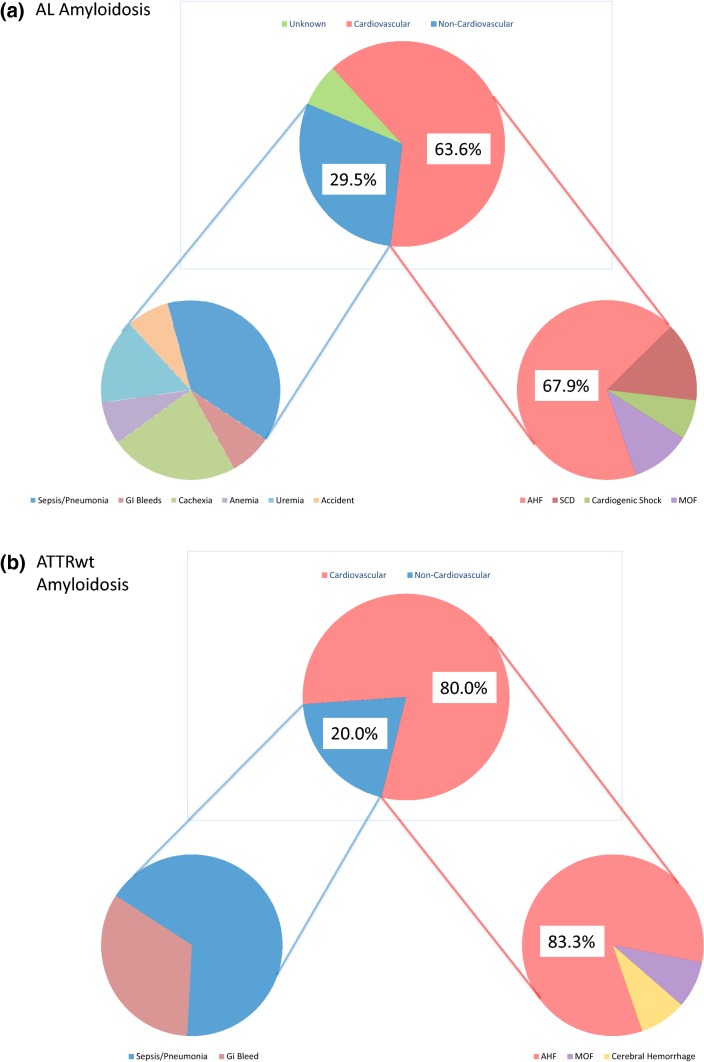
**a**, **b** Mode of death stratified by amyloid subtype. All deaths are divided into cardiovascular (CV), non-CV, and unknown deaths; cardiovascular deaths are further subdivided into sudden cardiac death (SCD), cardiogenic shock, acute heart failure, cerebral haemorrhage, and multi-organ failure (MOF), while non-CV deaths are subdivided into sepsis/pneumonia, uraemia, GI bleeding, anaemia, accident, and cachexia

Information on MOD was available for 56 (94.9%) of the deceased patients. Of the deceased patients 37 (62.7%) died in hospital, death was witnessed in another 9 (15.3%) patients. Autopsy was performed in 13 (22.0%) patients. Overall, MOD in (Fig. 3) was found to be CV in 40 (67.8%) and non-CV in 16 (27.1%) patients [sepsis in 4 (6.8%), pneumonia in 3 (5.1%), uraemia in 2 (3.4%), GI bleeding in 2 (3.4%), anaemia in 1 (1.7%), accident in 1 (1.7%), and cachexia in 3 (5.1%) patients, respectively], but remained unknown in 3 (5.1%) patients (AL) in the entire cohort. In deceased patients time to death was numerically, but not statistically, significantly shorter for CV [23.3 months (4.6–34.6)] compared with non-CV death events [26.8 months 4.7–34.0)] (*p* = 0.67). Almost all CV deaths were cardiac (97.5%) and one was vascular due to cerebral hemorrhage).

Among patients with documented MOD, CV [28 (68.3%) vs. 12 (80.0%)] and non-CV [13 (31.7%) vs. 3 (20.0%)] death events were distributed equally between AL and TTR subtypes (*p* = 0.513) (Fig. 3). Even when multi-organ failure (MOF) was considered non-CV, CV deaths still accounted for 61% in AL and 77.3% in TTR patients (*p* = 0.49). Also, there were no major differences between AL and TTR patients in terms of sudden cardiac death [4 (9.1%) vs. 0 (0.0%)], cardiogenic shock [2 (4.5%) vs. 0 (0.0%)], acute heart failure [19 (43.2%) vs. 10 (66.7%)], MOF [3 (6.8%) vs. 1 (6.7%)], sepsis/pneumonia [5 (11.4%) vs. 2 (13.3%)], uraemia [2 (4.5%) vs. 0 (0.0%)], GI bleeding [1 (2.3%) vs. 1 (6.7%)], anaemia [1 (2.3%) vs. 0 (0.0%)], accident [1 (2.3%) vs. 0 (0.0%)], and cachexia [3 (6.8%) vs. 0 (0.0%)] (Fig. 3).

## Discussion

This study describes a well-characterized cohort of patients with cardiac amyloidosis. Our study shows that although clinical presentation and disease progression differed between AL and ATTRwt, CV death was the predominant mode of death in both subtypes.

Mortality was high in the entire cohort of patients. Prognosis was significantly worse in AL than in ATTRwt. This is well in line with previous studies in patients with CA [2, 8, 13, 14]. Five-year mortality rate in our cohort was 65% in AL and 44% in TTR. The 25% 30-month mortality rate in the ATTR group corresponds largely to the number reported in the recently published ATTR-ACT study [25]. Although non-survivors presented at baseline with more severe heart failure symptoms effects of the amyloid subtype on mortality were independent of substantial confounders including NT-proBNP levels. Mayo staging scores were not different between survivors and non-survivors.

Information on MOD was available in 95% of patients. Similar to previous reports concerning heart failure [6, 24] 2.7% of our patients died in hospital. Necropsy was performed in a minority (22%) of patients. Interestingly, cardiovascular events accounted for more than two-thirds of fatal casualties in both groups. Circulatory failure comprising cardiogenic shock, acute heart failure and multi-organ failure was the predominant MOD. Thus, our data on MOD for the first time provide evidence for the predominance of CV deaths in patients with CA irrespective of the amyloid subtype, which was previously discussed in the literature [16]. MOD in our cohort of patients with cardiac amyloidosis agrees well with 60–70% CV deaths and 20–30% non-CV deaths, which were recently reported in patients with HFpEF of various aetiologies [26].

Since information on circumstances of death was retrieved retrospectively, exact definition of cause of death (COD) was difficult to determine in a substantial number of the deceased. Therefore, we refrain from reporting separately on COD. In contrast, MOD was able to be defined in most patients. Due to the systemic nature of the disease in AL with concomitant involvement of several organ systems, adjudication of MOD was complex in patients with MOF. Nevertheless, if patients with MOF were classified non-CV instead of CV, the proportion of circulatory failure events remained large. Acute heart failure was the main cause of CV deaths in AL and ATTRwt (68.3% and 80%, respectively). Similarity between groups in this regard is remarkable since first presentation of CA was different in both subtypes. The majority of ATTRwt patients presented with heart failure with preserved ejection fraction (HFpEF) characterized by greater LV mass and a larger percentage of atrial enlargement and atrial fibrillation compared to AL. In contrast, AL patients were younger with a female predominance, higher NYHA functional class and lower blood pressure, and a larger percentage of proteinuria. Discrepancies between groups in our cohort of CA patients are corroborated by comparable data from the existing literature [2, 7, 13, 14, 27, 28]. It is speculated that differences in extracellular amyloid deposition and a likely toxic effect on cardiomyocytes and their architecture, as demonstrated in AL amyloidosis [29], account for differences in phenotypic appearance and—together with non-cardiological factors—also for differences in mortality [2, 9].

In fact, our data show that heart failure is the predominant MOD in both subtypes despite significant differences in patient presentation and disease progression.

Interestingly, SCD was registered only in a minority of patients in both groups. It must be acknowledged that 25% of our patients were treated with an ICD and/or pacemaker. However, no appropriate ICD discharge was documented in these patients. Indeed, previous studies could not show a survival benefit despite appropriate ICD shocks in patients with CA [30, 31]. This was mostly attributed to electromechanical dissociation, as the underlying mechanism for SCD in CA.

The most common non-CV MOD in both subtypes was sepsis and pneumonia in 11.4% of AL and 13.3% of ATTRwt patients. The relatively small number in AL with the majority of patients treated with chemotherapy and/or autologous stem cell transplantation is contrary to common expectation. This phenomenon may indicate excellent oncologic patient management and emphasizes the need for interdisciplinary management of CA patients.

Of note, only one vascular death was registered in the entire cohort. This is remarkable as cardioembolic events and venous thromboembolism are frequent in amyloidosis [32]. In an early necropsy series, 26% of patients with CA had one or more cardiac chamber thrombi [33]. In our cohort, 28.2% of patients were in atrial fibrillation and thus particularly prone to cardioembolic events. It can be speculated that low incidence of deadly vascular events was due to a large percentage of patients on anticoagulant therapy in both subtypes. Intensive anticoagulant therapy, however, may have been penalized by three cases of deadly bleedings.

Importantly, disease prognosis has been altered with the development of new strategies that efficiently supress secretion of amyloid-forming light chains in AL and also with earlier diagnosis that may prevent irreversible damage to the heart [8, 15]. Up to now, treatment in ATTRwt was limited mostly to supportive care. The recent emergence of novel therapeutics such as tafamidis [25], which act to prevent transthyretin amyloid formation, and other agents that inhibit transthyretin expression [34, 35] offers promise for the near future in the management of ATTRwt, which probably constitutes the majority of CA patients. This underscores even more the cardiologists’ responsibilities with regard to early and exact diagnosis as well as comprehensive disease management strategies.

## Strengths and limitations

Strengths of our study include the comprehensive clinical characterization and complete follow-up of our sample. However, some limitations apply to this study. Information on COD, which could not be retrieved in a substantial number of patients, would have improved the value of the study. MOD in ten patients whose dead was not witnessed and who had no autopsy was classified based on death records a/o information received from relatives concerning the last hours of the deceased individuals. No information was available on non-deadly strokes, heart failure admissions, and pacemaker dependency, which would have improved assessment of disease progression.

## Conclusions

This analysis shows no differences in MOD between patients with AL and TTRwt cardiac amyloidosis despite significant differences in clinical presentation and disease progression. Cardiovascular events account for two-thirds of fatal causalities in both groups. Our findings underline the great responsibility of cardiologists in the management of CA and call for early diagnosis, meticulous fluid control to avoid congestion, and prevention or even reversal of disease progression by providing vigorous treatment of the underlying pathomechanism by an interdisciplinary team.
